# Complete mitochondrial genome of *Bactrocera arecae* (Insecta: Tephritidae) by next-generation sequencing and molecular phylogeny of Dacini tribe

**DOI:** 10.1038/srep15155

**Published:** 2015-10-16

**Authors:** Hoi-Sen Yong, Sze-Looi Song, Phaik-Eem Lim, Kok-Gan Chan, Wan-Loo Chow, Praphathip Eamsobhana

**Affiliations:** 1 Institute of Biological Sciences, University of Malaya, 50603 Kuala Lumpur, Malaysia; 2 Chancellery High Impact Research, University of Malaya, 50603 Kuala Lumpur, Malaysia; 3 Institute of Ocean and Earth Sciences, University of Malaya, 50603 Kuala Lumpur, Malaysia; 4 Science Vision Sdn Bhd, Setia Avenue, 33A-4 Jalan Setia Prima S, U13/S, Setia Alam, Seksyen U13, 40170 Shah Alam, Selangor Darul Ehsan, Malaysia; 5 Department of Parasitology, Faculty of Medicine Siriraj Hospital, Mahidol University, Bangkok 10700, Thailand

## Abstract

The whole mitochondrial genome of the pest fruit fly *Bactrocera arecae* was obtained from next-generation sequencing of genomic DNA. It had a total length of 15,900 bp, consisting of 13 protein-coding genes, 2 rRNA genes, 22 tRNA genes and a non-coding region (A + T-rich control region). The control region (952 bp) was flanked by *rrnS* and *trnI* genes. The start codons included 6 ATG, 3 ATT and 1 each of ATA, ATC, GTG and TCG. Eight TAA, two TAG, one incomplete TA and two incomplete T stop codons were represented in the protein-coding genes. The cloverleaf structure for *trnS1* lacked the D-loop, and that of *trnN* and *trnF* lacked the TΨC-loop. Molecular phylogeny based on 13 protein-coding genes was concordant with 37 mitochondrial genes, with *B. arecae* having closest genetic affinity to *B. tryoni*. The subgenus *Bactrocera* of Dacini tribe and the Dacinae subfamily (Dacini and Ceratitidini tribes) were monophyletic. The whole mitogenome of *B. arecae* will serve as a useful dataset for studying the genetics, systematics and phylogenetic relationships of the many species of *Bactrocera* genus in particular, and tephritid fruit flies in general.

Some 200 species of tephritid fruit flies in the world are considered pests of economic importance, causing direct losses to a wide variety of fruit, vegetable and flower crops^1^. The larvae of about 35% of the species attack soft fruits^2^. Members of the Dacini tribe, particularly the *Bactrocera* genus, are of special economic importance in tropical Asia, Australia and the South Pacific^2^.

The betelnut fruit fly *Bactrocera arecae* (Hardy & Adachi) is a member of the *Bactrocera dorsalis* species complex of the Dacinae subfamily^1^,^3^. It has a predominantly orange to brown body, with a broad yellow median band on the mesonotum. Its larvae feed in the nuts of the betelnut palm (*Areca catechu*)^3^. The adult male flies are not attracted to Cue lure or methyl eugenol^2^. This species has a restricted distribution, from southern Thailand through Peninsular Malaysia to Singapore (the type locality)^1^,^3^.

To date, *B. arecae* has not received extensive attention in molecular phylogenetic studies. For example, it was not included in Smith *et.al*.’s study on the phylogenetic relationships among 24 *Bactrocera* species based on *rrnL*, *cox2*, *trnK* and *trnD* genes^4^, and Krosh *et.al*.’s study of 125 Dacini species based on *rrnL*, *cox1*, *cox2* and “white-eye” genes^5^. More recently, it was among 47 *Bactrocera* species studied based on *cox1* gene sequences^6^, and Virgilio *et al*.’s study of 56 *Bactrocera* taxa using *cox1* and *rrnL* gene fragments^7^. To date there are only 7 nucleotide sequences for *B. arecae* in GenBank – 2 for *cox1* and one each for ITS-1, *cox2*, *nad1*, *rrnL* and *rrnS*. At the mitochondrial genome (mitogenome) level, only 10 whole genomes of *Bactrocera* taxa are available in GenBank.

We report here the whole mitogenome of *B. arecae* determined using next-generation sequencing (NGS) and discuss the molecular phylogeny of Dacini tribe.

## Results

### Mitogenome analysis and features

Next-generation sequencing on NextSeq 500 Dekstop Sequencer generated an approximately 100 giga bases data from *B. arecae* library. Removal of low quality sequence (<Q20), PhiX reads and sequences shorter than 50 nucleotides resulted in 459 million paired-end reads with 57 billion bases. A total of 2,451,320 sequence reads with a total of 330,821,510 bases were mapped to full mitochondrial genome reference sequences of *Bactrocera* genus. *De novo* assembly of these mapped reads resulted in 164 contigs with maximum length of 15,925 bp and N50 of 485. The total GC content was 28.4%, with base composition of 33.8% A, 37.9% T, 17% G, and 11.4% C.

**Figure 1:**
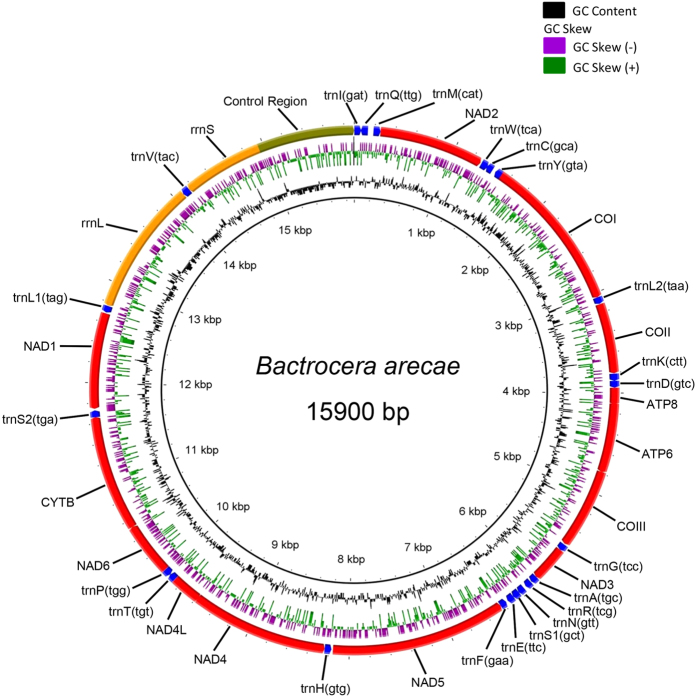
Complete mitogenome of *Bactrocera arecae* with BRIG visualization showing the protein coding genes, rRNAs, tRNAs and non-coding regions. GC skew (−0.259) is shown on the outer surface of the ring whereas GC content (27.7%) is shown on the inner surface. The AT skew is 0.080.

The mitogenome of *B. arecae* was 15,900 bp long, comprising 37 genes (13 protein-coding genes – PCGs, 2 rRNA genes, and 22 tRNA genes) and a non-coding region (A + T-rich control region) (Table 1, Fig. 1). Spacing sequences ranged from 2 to 55 bp in 15 regions, the largest was between *trnQ* and *trnM* genes. Sequences with 25, 33 and 55 bases had clear stem-loop structures (Supplementary Fig. S1). The overlaps in 9 regions ranged from 1 to 8 bp, the largest being between *trnW* and *trnC* genes (Table 1). Nine PCGs (*nad2*, *cox1-3*, *atp6*, *atp8*, *nad3*, *nad6*, *cob*), 14 tRNAs and the control region were located on the major J-strand (Table 1). Fourteen genes (4 PCGs – *nad5, nad4, nad4l* and *nad1*; both rRNA genes; and 8 tRNA genes) were located on the minor N-strand. The control region (952 bp) was flanked by *rrnS* and *trnI* genes (Fig. 1). It conformed to the general structure in insects, comprising two poly-T stretches with ≥7 bp (one with 25 bp and the other 7 bp). There were 7 T-stretch with 4 bp, 2 with 5 bp and 1 with 6 bp.

The commonest start codon was ATG (in 6 PCGs – *cox2*, *atp6*, *cox3*, *nad4l*, *nad4*, *cob*), followed by three for ATT (*nad2*, *nad3*, *nad6*), and one each for ATA (*nad1*), ATC (*nad5*), GTG (*atp8*) and TCG (*cox1*). Eight PCGs had TAA stop codon, two had TAG while the remaining three genes had incomplete stop codons (TA in *cox1*; T in *nad1* and *nad3*) (Table 1).

Table 2 summarizes the base composition of the mitochondrial whole genome, protein-coding genes, rRNA genes and control region. All were A + T rich. The A + T content for PCGs ranged from 63.3% (*cox1*) to 78.8% (*nad4l*). Eight PCGs (*atp8* and all 7 *nad*) had A + T content of over 70%. The A + T content of the non-coding control region was 86.0%. The GC skewness values for the whole genome, PCGs, rRNA genes and control region were negative (−0.120 to −0.487) indicating bias toward the use of Cs over Gs. Although the AT skewness value was positive (0.080) for the whole genome, it was variable for individual genes.

**Figure 2:**
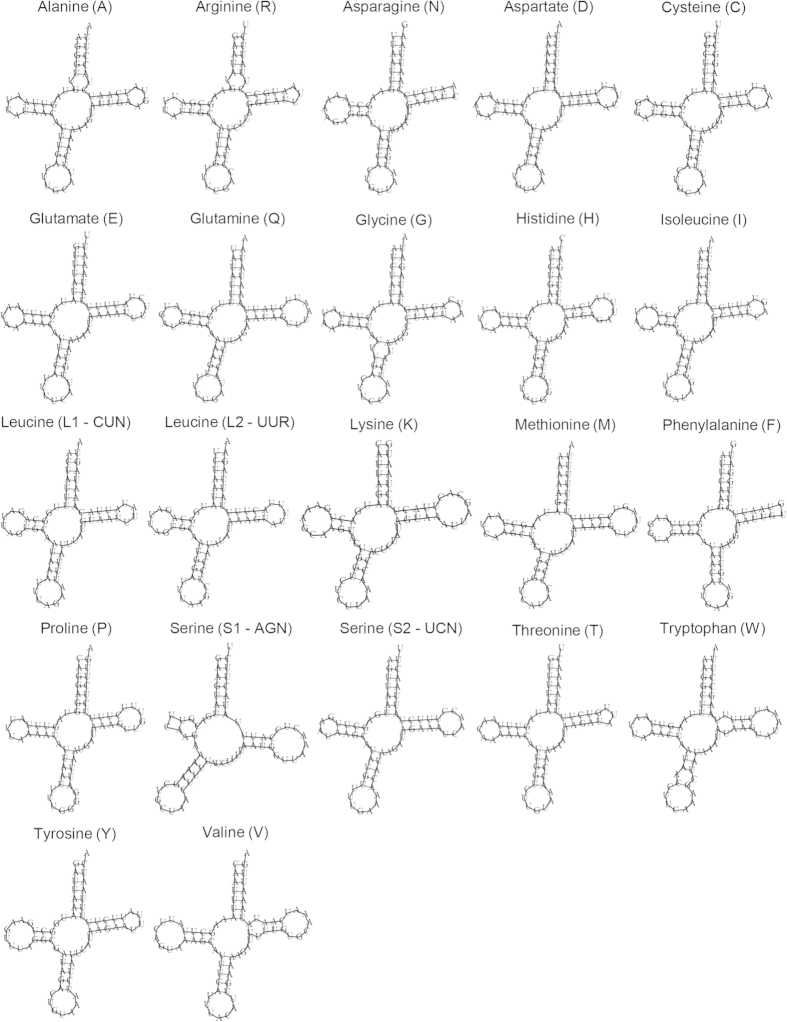
Cloverleaf structure of the 22 inferred tRNAs in the mitogenome of *Bactrocera arecae*. The cloverleaf structure for *trnS1* lacked the D-loop, and that of *trnN* and *trnF* lacked the TΨC-loop.

Of the tRNAs, the cloverleaf structure for *trnS1* lacked the D-loop, while *trnN* and *trnF* lacked the TΨC-loop (Fig. 2). The number of base pairs in the DHU-stem ranged from 3 to 4 (Fig. 2; Supplementary Table S1). All the TΨC-stems had 5 base pairs except 4 bp in *trnC*, *trnH*, *trnP* and *trnS1*. The number of bases in the D-loop and TΨC-loop was variable.

### Phylogenetic relationships within Dacini tribe

**Figure 3:**
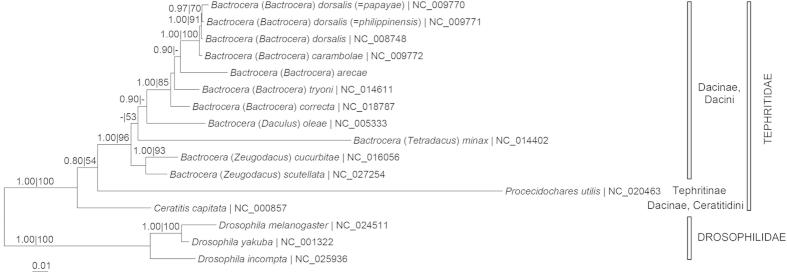
Bayesian inference and maximum likelihood tree based on 13 protein-coding genes of the whole mitogenomes of Tephritid fruit flies with Drosophilidae as outgroup. Numeric values at the nodes are Bayesian posterior probabilities/ML bootstrap.

**Figure 4:**
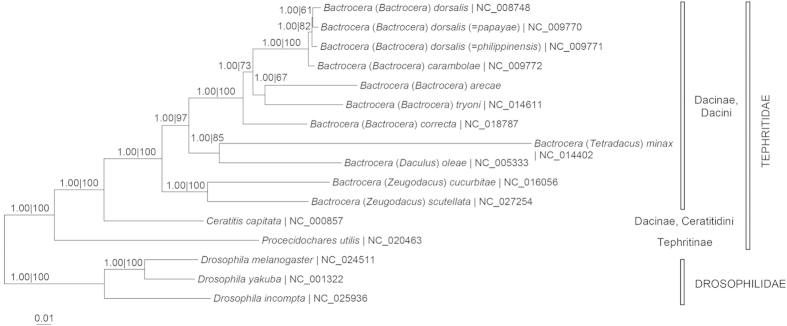
Bayesian inference and maximum likelihood tree based on *rrnL* and *rrnS* genes from whole mitogenomes of Tephritid fruit flies with Drosophilidae as outgroup. Numeric values at the nodes are Bayesian posterior probabilities/ML bootstrap.

Figure 3 depicts the molecular phylogeny of *B. arecae* in relation to other taxa of the Dacini tribe of Dacinae subfamily based on 13 PCGs. The phylogram based on 37 mt-genes (13 PCGs, 2 rRNA and 22 tRNA genes) was congruent with that based on 13 PCGs (Supplementary Fig. S2). Most of the nodes were well-supported. The genus *Bactrocera* was monophyletic. Members of the subgenus *Bactrocera* formed a distinct clade from the other subgenera (*Daculus*, *Tetradacus* and *Zeugodacus*), with *B*. (*B*.) *arecae* forming a sister group to *B*. (*B*.) *tryoni*. The subfamily Dacinae was also monophyletic and clearly separated from Tephritinae subfamily. However, based on *rrnL* and *rrnS* genes the subfamily Dacinae was not monophyletic as *Procecidochares utilis* (Tephritinae subfamily) was sister to the Dacini (Fig. 4).

## Discussion

Mitochondrial genomes of insects have been very extensively studied^8^. They have been applied particularly to studies regarding phylogeny and evolution^8^. To date there are 12 complete mitogenomes of tephritid fruit flies in GenBank – 10 from *Bactrocera* (Dacinae, Dacini), and 1 each from *Ceratitis* (Dacinae, Ceratitidini) and *Procecidochares* (Tephritinae, Cecidocharini).

The mitogenome size of *B. arecae* (15,900 bp) is smaller than those of *B. carambolae* (15,915 bp), *B. correcta* (15,936 bp), *B. dorsalis* (15,915 bp), *B. minax* (16,043 bp), *B. dorsalis* (= *papayae*) (15,915 bp), *B. dorsalis* ( = *philippinensis*) (15,915 bp), *B. scutellata* (15,915 bp), *B. tryoni* (15,925 bp), *C. capitata* (15,980 bp) and *P. utilis* (15,922 bp) but larger than those of *B. oleae* (15,815 bp) and *B. cucurbitae* (15,825 bp).

The A + T content of the control region in *B. arecae* (86.0%) is higher than those of *B. cucurbitae* (82.4%), *B. correcta* (78.6%), *B. minax* (77.6%) and *B. scutellata* (72.6%) but lower than those of *B. oleae* (86.9%), *B. tryoni* (87.0%), *B. carambolae* (87.9%), *B. dorsalis* (88.1%), *B. dorsalis* (=*papayae*) (88.2%), *B. dorsalis* (=*philippinensis*) (88.2%) and *C. capitata* (91.1%).

In *B. arecae* mitogenome, in addition to 8 TAA, 2 TAG, and 3 incomplete (1 TA and 2 T) stop codons are represented in the protein-coding genes (Table 1). This differs from some tephritid mitogenomes which possess additionally truncated TA stop codon (e.g. *B. dorsalis*, *B. oleae*)^9^,^10^ or TAT stop codon (*B. minax*)^11^. Among the tephritid fruit flies, over half of the PCGs in *B. dorsalis* have truncated stop codons (3 TA and 4 T)^9^. The incomplete T–stop codons can be converted to TAA by post-translational polyadenylation^12^. Additionally, the *nad5* gene of *B. arecae* has ATC instead of ATT start codon found in congeners (*B. dorsalis*, *B. minax*, *B. oleae*).

As in other insects, the *B. arecae* mitogenome has three main clusters of characteristic tRNAs (Fig. 1): (1) I-Q-M (isoleucine, glutamate and methionine); (2) W-C-Y (tryptophan, cysteine and tyrosine); and (3) A-R-N-S1-E-F (alanine, arginine, asparagine, serine S1, glutamate and phenylalanine). The atypical cloverleaf structure of *trnS1* is common in all Metazoa^13^.

Our finding of *B. arecae* forming a sister group with *B. tryoni* and *B. minax* with *B. oleae* based on 13 PCGs and 37 mt-genes (Fig. 3, Supplementary Fig. S2) concurred with the phylogeny based on *cox1* sequences^6^. However, based on 13 PCGs and 37 mt-genes the sister group of *B. arecae* plus *B. tryoni* was sister to the *B. dorsalis* lineage. *B. correcta* was sister to the *B. dorsalis* lineage based on one study of *cox1* sequences^6^. Other studies based of *cox1*^14^ and multi-gene sudies^5^ also indicated closer affinity of *B. arecae* and/or *B. tryoni* to the *B. dorsalis* complex than of *B. correcta* to *B. dorsalis*. However, the analysis based on concatenated *cox1* and *rrnL* sequences indicated *B. tryoni* to be closer to *B. dorsalis* complex while *B. arecae* and *B. correcta* belonged to different lineages^7^.

The phylogenetic relationships of *B. dorsalis*, *B. dorsalis* (=*papayae*) and *B. dorsalis* (=*philippinensis*) based on different genetic markers were not congruent. Our analysis based on 13 PCGs and 37 mt-genes indicated *B. dorsalis* and *B. dorsalis* (=*papayae*) as sister taxa (Fig. 3, Supplementary Fig. S2) which agreed with that based on *cox1* and *cox2* sequences^15^ as well as *cox1*, *rrnL*, *trnP*, *nad6* and period sequences^7^ but differed from that based on *cox1* sequences which indicated *B. dorsalis* and *B. dorsalis* (=*philippinensis*) as sister taxa^6^, and *B. dorsalis* (=*papayae*) and *B. dorsalis* (=*philippinensis*) as sister taxa based on 37 mitochondrial genes^16^ and *rrnL*, *cox1*, *cox2* and “white-eye” genes^5^. In an earlier study based on 13 PCGs but included only 7 *Bactrocera* species^17^, *B. dorsalis* (=*papayae*) formed a sister group with *B. dorsalis* (=*philippinensis*) compared to *B. dorsalis*. Our study included *B. arecae*, *B. correcta*, *B. cucurbitae*, and *B. scutellata* as well as *P. utilis* (Tephritinae subfamily). The phylogeny based on *rrnL* and *rrnS* genes in our study also indicated closer affinity of *B. dorsalis* (=*papayae*) and *B. dorsalis* (=*philippinensis*) compared to *B. dorsalis* (Fig. 4). It is evident that gene markers form a contributory factor to the discrepancies of these results. The phylogeny based on 17 enzyme loci indicated close genetic affinity (Nei’s I = 0.99; D = 0.01) between *B. dorsalis* and *B. dorsalis* (=*papayae*)^18^. Based on our present analysis of 13 PCGs, the uncorrected genetic ‘p’-distance is 1.06 between *B. dorsalis* and *B. dorsalis* (=*papayae*) and 1.11 between *B. dorsalis* and *B. dorsalis* ( = *philippinensis*). A recent study based on six loci (*cox1*, *nad4*-3′, CAD, *period*, ITS1, ITS2) indicates that *B. dorsalis s.s*., *B. papayae* and *B. philippinensis* are the same biological species^19^. Another taxon *B. invadens* has also been synonymized with *B. dorsalis*^20^.

As in most other studies^6^,^7^,^16^,^17^,^18^,^19^, *B. carambolae* is closely related but distinct from *B. dorsalis*, *B. dorsalis* (=*papayae*) and *B. dorsalis* (=*philippinensis*) (Fig. 3, Supplementary Fig. S2). In the study based on *rrnL*, *cox1*, *cox2* and “white-eye” genes, *B. carambolae* was closer related to *B. invadens*, *B. dorsalis* (=*papayae*) and *B. dorsalis* (=*philippinensis*) than *B. dorsalis*^5^. Based on 17 enzyme loci, *B. carambolae* has a genetic identity of I = 0.92 (genetic distance of 0.08) compared to *B. dorsalis* and *B. dorsalis* (=*papayae*)^18^. Our present study based on 13 PCGs indicates a genetic distance of ‘p’ = 1.39 between *B*. *carambolae* and *B. dorsalis*, ‘p’ = 1.21 between *B. carambolae* and *B. dorsalis* (=*papayae*), and ‘p’=1.19 between *B. carambolae* and *B. dorsalis* (=*philippinensis*).

In the present study, *B*. (*Zeugodacus*) *cucurbitae* and *B*. (*Zeugodacus*) *scutellata* are related but distinct from the other subgenera (*Bactrocera*, *Daculus* and *Tetradacus*) of *Bactrocera* genus (Fig. 3, Supplementary Fig. S2). It has been proposed, based on *rrnL*, *cox1*, *cox2* and “white-eye” genes, that taxonomic consideration be given to raising *Zeugodacus* to genus level as the ‘*Zeugodacus*’ clade is the sister group to *Dacus*, not *Bactrocera*^5^. This was supported by the analysis based on *cox1*, *rrnL*, *trnP*, *nad6* and period sequences^7^. However, in the study based on *rrnL*, *cox1*, *cox2* and “white-eye” genes^5^, *Anastrepha ludens* and *Rhagoletis pomonella* (both are members of Trypetinae subfamily) were closer to Dacini (Dacinae subfamily) than to *C. capitata*. In the present study, the Ceratitidini tribe forms the sister group of the Dacini tribe (Fig. 3, Supplementary Fig. S2). The Tephritinae subfamily (represented by *P. utilis*) is distinct from Dacinae subfamily. This concurs with the finding of monophyly for tephritid subfamilies and tribes (Trypetini, Carpomyini, Tephritinae, and Dacinae) based on *rrnS*, *rrnL*, and *cox2* gene sequences^21^. In contrast, the phylogeny based on *rrnL* and *rrnS* genes indicated closer affinity of *P. utilis* to Dacini tribe than *C. capitata* (Fig. 4). A broader taxon sampling, particularly mitogenomes of *Dacus* genus (Dacini tribe), Tephritinae and Trypetinae subfamilies, is needed to resolve the higher order phylogeny.

In summary, we have successfully sequenced the whole mitochondrial genome of *B. arecae* by next-generation sequencing. The genome features are similar to other tephritid fruit flies except the ATC start codon for *nad5* gene and the absence of TΨC-loop in *trnN* and *trnF* tRNAs. The phylogenetic tree based on 13 PCGs is concordant with that based on 37 mt-genes. *B. arecae* shows closest genetic affinity to *B. tryoni*.

## Methods

### Ethics statement

*Bactrocera arecae* is not a protected or endangered species. No permission is needed to collect and study this fruit fly, a pest of *A. catechu*.

### Specimen collection

Fallen nuts of *A. catechu* were collected from the University of Malaya campus. They were placed in an aquarium with suitable substrate for the larvae to hatch. Pupae were placed in plastic tubes and emerging adults were collected, preserved in absolute ethanol and stored in deep freezer until use.

### Extraction of genomic DNA

Genomic DNA was extracted from thorax and legs using G-spin^TM^ Total DNA Extraction Mini Kit (iNtRON Biotechnology, Inc, Korea) following the manufacturer’s instructions with minor modification.

### Sample and library preparation

The purified genomic DNA was quantified with Qubit® 2.0 Fluorometer (Life Technologies, USA) and normalized to 2 μg. The normalized genomic DNA was fragmented to an average size of 550 bp using Covaris M220 system (Covaris, Woburn, MA, USA). Library was prepared using TruSeq DNA PCR-Free Sample Preparation Kit (Illumina, USA) following the manufacturer’s protocols. Quantification and size estimation of the library was conducted on a 2100 Bioanalyzer using High Sensitivity DNA Analysis Kit (Agilent Technologies) and quantitative real-time PCR was performed using KAPA Library Quantification Kit for Illumina sequencing platforms (KAPA Biosystems, Boston, MA, USA) on Eco Real-Time PCR System.

### Genome sequencing

The library was normalized to 1.5 pM and sequenced using the NextSeq 500 Dekstop Sequencer (2 × 150 bp paired-end reads) (Illumina, USA).

### Sequence and genome analysis

Raw sequences were extracted from the Illumina NextSeq 500 system in FASTQ format and the quality of sequences was evaluated using the FastQC software^22^. All the ambiguous nucleotides and reads with an average quality value (lower than Q20) were excluded from further analysis. The trimmed sequences were mapped against three reference mitogenomes, namely, *Bactrocera dorsalis* (NC_008748), *Bactrocera carambolae* (NC_009772) and *Bactrocera dorsalis* (=*papayae*) (NC_009770) using the CLC Genomic Workbench v.7.0.4 (Qiagen, Germany). The mapped sequences were then subjected to *de novo* assembly. High coverage contigs (average coverage value more than 500) greater than 15 kbp were subjected to BLAST^23^ alignment against the nucleotide database at National Center for Biotechnology Information (NCBI). Contigs with hits to mitochondrial genes or genomes were identified and extracted from CLC Genomic Workbench.

### Mitogenome identification and annotation

A contig identified as mitogenome was manually examined for repeats at the beginning and end of the sequence to establish a circular mtDNA. It was then annotated with MITOS^24^ followed by manual validation of the coding regions using the NCBI ORF Finder (http://www.ncbi.nlm.nih.gov/gorf/gorf.html). The sequin file generated from MITOS was edited and submitted to NCBI according to ORF Finder result (NCBI GenBank accession number KR233259).

### Mitogenome visualization

The circular mitogenome of *B. arecae* was visualized with Blast Ring Image Generator (BRIG)^25^.

### Phylogenetic analysis

Mitogenome sequences (12 taxa) of Tephritidae family that were available in GenBank were used with *B. arecae* to reconstruct phylogenetic trees (Figs 3 and 4; Supplementary Fig. S1). *Drosophila incompta* NC_025936, *D. melanogaster* NC_024511 and *D. yakuba* NC_001322 of the Drosophilidae family were included as outgroups.

Nucleotide sequences of the 13 protein-coding genes (PCGs) were separately aligned using ClustalX v.1.81^26^ program and subsequently edited and trimmed using BioEdit v.7.0.5.3^27^. The sequences of *rrnS*, *rrnL* and 22 mt-tRNA genes were aligned by MAFFT v.7^28^. The best-fit nucleotide substitution models for maximum likelihood (ML) using the corrected Akaike Information Criterion^29^ and Bayesian (BI) analyses using the Bayesian Information Criterion^30^ were determined by Kakusan v.3^31^. Phylograms of 13 concatenated PCGs, 37 mt-genes and 2 rRNA genes were constructed using TreeFinder^32^. Bootstrap values (BP) were generated via 1,000 ML bootstrap replicates. Bayesian analyses were conducted using the Markov chain Monte Carlo (MCMC) method via Mr. Bayes v.3.1.2^33^, with two independent runs of 2 × 10^6^ generations with four chains, and with trees sampled every 200^th^ generation. Likelihood values for all post-analysis trees and parameters were evaluated for convergence and burn-in using the “sump” command in MrBayes and the computer program Tracer v.1.5 (http://tree.bio.ed.ac.uk/software/tracer/). The first 200 trees from each run were discarded as burn-in (where the likelihood values were stabilized prior to the burn-in), and the remaining trees were used for the construction of a 50% majority-rule consensus tree. Phylogenetic trees were viewed and edited by FigTree v.1.4^34^,^35^,^36^

## Additional Information

**Accession Code:** *Bactrocera arecae* mitogenome sequences are available in NCBI GenBank database (accession number: KR233259).

**How to cite this article**: Yong, H.-S. *et al*. Complete mitochondrial genome of *Bactrocera arecae* (Insecta: Tephritidae) by next-generation sequencing and molecular phylogeny of Dacini tribe. *Sci. Rep*. **5**, 15155; doi: 10.1038/srep15155 (2015).

## Supplementary Material

Supplementary Information

## Figures and Tables

**Table 1:** Characteristics of the mitochondrial genome of *Bactrocera arecae*.

Gene	Location	Strand	Size (bp)	IntergenicSequence	Start/stopcodon
*trnI*(gat)	1–66	J	66	−3	
*trnQ*(ttg)	64–132	N	69	55	
*trnM*(cat)	188–256	J	69		
*nad2*	257–1279	J	1023	12	ATT/TAA
*trnW*(tca)	1292–1360	J	69	−8	
*trnC*(gca)	1353–1416	N	64	33	
*trnY*(gta)	1450–1516	N	67	−2	
*cox1*	1515–3049	J	1535		TCG/TA
*trnL2*(taa)	3050–3115	J	66	4	
*cox2*	3120–3809	J	690	3	ATG/TAA
*trnK*(ctt)	3813–3882	J	70		
*trnD*(gtc)	3883–3949	J	67		
*atp8*	3950–4111	J	162	−7	GTG/TAA
*atp6*	4105 –4782	J	678	−1	ATG/TAA
*cox3*	4782–5570	J	789	9	ATG/TAA
*trnG*(tcc)	5580–5644	J	65		
*nad3*	5645–5996	J	352		ATT/T
*trnA*(tgc)	5997–6061	J	65	7	
*trnR*(tcg)	6069–6132	J	64	25	
*trnN*(gtt)	6158–6222	J	65		
*trnS1*(gct)	6223–6290	J	68		
*trnE*(ttc)	6291–6357	J	67	18	
*trnF*(gaa)	6376–6440	N	65	5	
*nad5*	6446–8158	N	1713	15	ATC/TAA
*trnH*(gtg)	8174–8239	N	66		
*nad4*	8240–9580	N	1341	−7	ATG/TAG
*nad4l*	9574–9870	N	297	2	ATG/TAA
*trnT*(tgt)	9873–9937	J	65		
*trnP*(tgg)	9938–10003	N	66	2	
*nad6*	10006–10530	J	525	−1	ATT/TAA
*cob*	10530–11666	J	1137	−2	ATG/TAG
*trnS2*(tga)	11665–11731	J	67	15	
*nad1*	11747–12686	N	940	10	ATA/T
*trnL1*(tag)	12697–12761	N	65	4	
*rrnL*	12766–14088	N	1323		
*trnV(*tac)	14089–14160	N	72		
*rrnS*	14161–14948	N	788		
Control region	14949–15900	J	952		

**Table 2:** Base composition of mitochondrial whole genome, protein-coding genes, rRNA genes and control region.

Region	A%	C%	G%	T%	A + T%	G + C%	AT skew	GC skew
Whole genome	39.0	17.5	10.3	33.2	72.3	27.7	0.080	−0.259
*nad2*	33.3	19.5	9.1	38.1	71.5	28.5	−0.067	−0.364
*cox1*	29.9	20.5	16.1	33.4	63.3	36.7	−0.055	−0.120
*cox2*	33.5	19.4	13.6	33.5	67.0	33.0	0	−0.176
*atp8*	35.2	20.4	9.2	35.2	70.4	29.6	0	−0.378
*atp6*	30.5	20.1	11.8	37.6	68.1	31.9	−0.104	−0.259
*cox3*	29.9	20.6	15.0	34.5	64.4	35.6	−0.071	−0.157
*nad3*	32.4	19.0	9.7	38.9	71.3	28.7	−0.091	−0.324
*nad5*	45.3	18.7	9.2	26.8	72.2	27.8	0.257	−0.341
*nad4*	48.5	16.7	8.5	26.3	74.8	25.2	0.297	−0.325
*nad4l*	51.2	14.8	6.4	27.6	78.8	21.2	0.299	−0.396
*nad6*	38.1	17.7	6.1	38.1	76.2	23.8	0	−0.487
*cob*	31.3	21.3	13.3	34.1	65.4	34.6	−0.043	−0.231
*nad1*	48.0	18.7	9.8	23.5	71.5	28.5	0.343	−0.312
*rrnS*	43.1	14.3	6.8	35.8	78.8	21.2	0.093	−0.355
*rrnL*	40.7	16.8	9.0	33.5	74.2	25.8	0.097	−0.302
Control region	45.6	7.9	6.1	40.4	86.0	14.0	0.060	−0.129
