# Supplementary material for: Complete mitochondrial genome of Bactrocera arecae (Insecta: Tephritidae) by next-generation sequencing and molecular phylogeny of Dacini tribe
Source: Sci Rep. 2015 Oct 16;5:15155. doi: 10.1038/srep15155 (PMC4607999; doi:10.1038/srep15155)
Supplement: Supplementary Information [file srep15155-s1.doc]

Complete mitochondrial genome of *Bactrocera arecae* (Insecta: Tephritidae) by next-generation sequencing and molecular phylogeny of Dacini tribe

Hoi-Sen Yong^1,2,*^, Sze-Looi Song^2^, Phaik-Eem Lim^3,*^, Kok-Gan Chan^1,2^, Wan-Loo Chow^4^ and Praphathip Eamsobhana^5^

^1^Institute of Biological Sciences, University of Malaya, 50603 Kuala Lumpur, Malaysia, ^2^Chancellery High Impact Research, University of Malaya, 50603 Kuala Lumpur, Malaysia, ^3^Institute of Ocean and Earth Sciences, University of Malaya, 50603 Kuala Lumpur, Malaysia, ^4^Science Vision Sdn Bhd,  Setia Avenue, 33A-4 Jalan Setia Prima S, U13/S, Setia Alam, Seksyen U13, 40170 Shah Alam, Selangor Darul Ehsan, Malaysia, ^5^Deaprtment of Parasitology, Faculty of Medicine Siriraj Hospital, Mahidol University, Bangkok 10700, Thailand

Supplementary Table S1 ▏ Number of base pairs in DHU-stem and TΨC-stem of mt-tRNAs of *Bactrocera arecae*.

| tRNA | DHU-stem | TΨC-stem |
| --- | --- | --- |
| Alanine | 4 | 5 |
| Arginine | 4 | 5 |
| Asparagine | 3 | 5^#^ |
| Aspartate | 4 | 5 |
| Cysteine | 4 | 4 |
| Glumate | 4 | 5 |
| Glutamine | 4 | 5 |
| Glycine | 4 | 5 |
| Histidine | 4 | 4 |
| Isoleucine | 3 | 5 |
| Leucine L1 (CUN) | 3 | 5 |
| Leucine L2 (UUR) | 3 | 5 |
| Lysine | 3 | 5 |
| Methionine | 4 | 5 |
| Phenyalanine | 4 | 5^#^ |
| Proline | 4 | 4 |
| Serine S1 (AGN) | 4* | 4 |
| Serine S2 (UCN) | 4 | 5 |
| Threonine | 4 | 5 |
| Tryptophan | 4 | 5 |
| Tyrosine | 3 | 5 |
| Valine | 4 | 5 |

*without D-loop; ^#^without TΨC-loop

**
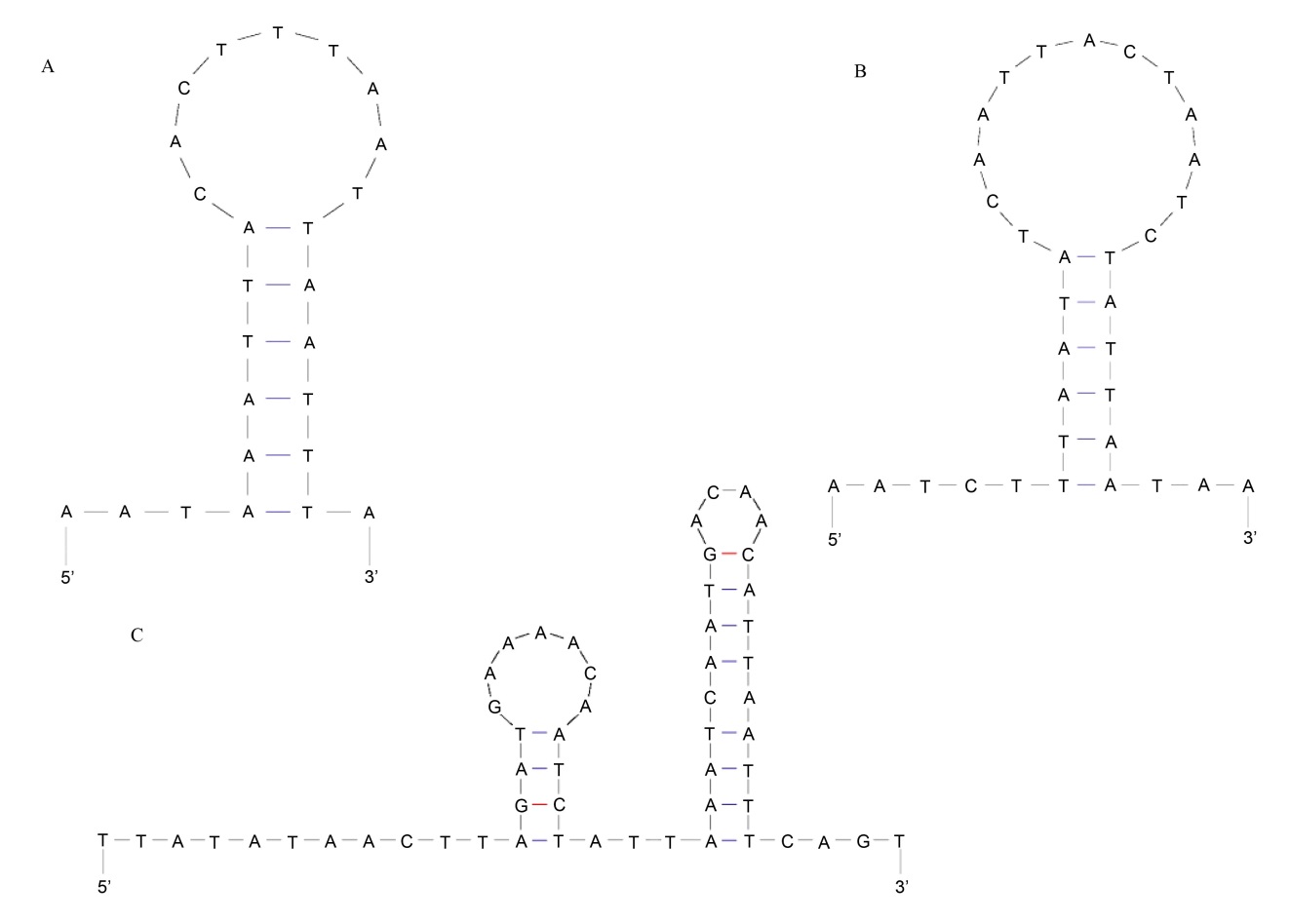
**

**Supplementary Figure S1 ▏ Stem-loop structure of intergenic spacing sequences of *Bactrocera arecae* mitochondrial genome.** A, 25 bases; B, 33 bases; C, 55 bases.


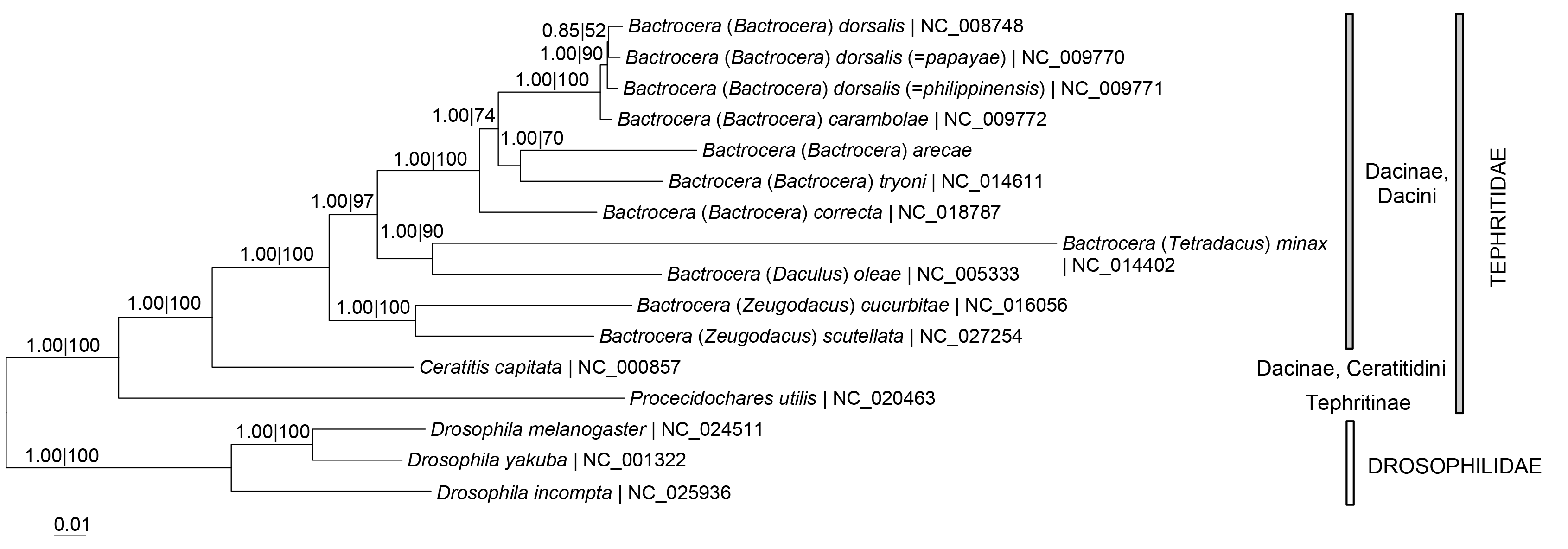


**Supplementary Figure S2 ▏ Bayesian inference and maximum likelihood tree based on 37 mitochondrial genes of the whole mitogenomes of Tephritid fruit flies with Drosophilidae as outgroup.** Numeric values at the nodes are Bayesian posterior probabilities/ML bootstrap.
